# Occurrences, human harm and years of life lost due to natural disasters in the state of Rio de Janeiro, Brazil, 2010-2022: a cohort study

**DOI:** 10.1590/S2237-96222025v34e20240412.en

**Published:** 2025-04-18

**Authors:** Roberta Fernanda da Paz de Souza Paiva, Ana Luiza de Oliveira Maia, Juliana Beloti Giarola Martins

**Affiliations:** 1 Universidade Federal Fluminense, Escola de Engenharia Industrial Metalúrgica de Volta Redonda, Programa de Pós-Graduação em Tecnologia Ambiental, Volta Redonda, RJ, Brazil; 2 Universidade Federal Fluminense, Escola de Engenharia Industrial Metalúrgica de Volta Redonda, Engenharia de Agronegócios, Volta Redonda, RJ, Brazil

**Keywords:** Climate Change, Premature Deaths, Life Expectancy, Disaster Vulnerability, Cohort Studies, Cambio Climático, Muertes Prematuras, Esperanza de Vida, Vulnerabilidad ante Desastres, Estudios de Cohortes

## Abstract

**Objective:**

To describe occurrences, human harm and material damage associated with natural disasters that occurred in the state of Rio de Janeiro, Brazil, 2010-2022, as well as to estimate years of life lost due to deaths resulting from these events.

**Methods:**

This is a prospective cohort study, with data from the Integrated Disaster Information System and the Brazilian National Health System Hospital Information System. Descriptive analyses of occurrences, human harm and material damage associated with the natural disasters selected and calculation of years of life lost due to deaths recorded under codes X36-39, by year, sex and age group.

**Results:**

752 extreme natural events occurred, with the highest frequency being observed for intense rainfall (37.8%) and mass movements (19.6%), followed by floods (15.3%), flash floods (15.2%), pluvial floods (8.9%) and hail (3.3%). As for human harm, 1,523 deaths, 3,379 injuries and 2,157 illnesses were recorded. Regarding material damage, the cost was calculated to be over BRL 12 billion, with housing units, health and education facilities and urban infrastructure being most affected. The majority of deaths were among females (50.1%). The 15-59 year age group was the most affected (51.8%). A total of 49,031.76 years of life were calculated to have been lost, of which 54.2% related to females.

**Conclusion:**

Natural disaster occurrence, human harm and material damage affected, more intensely, only a few municipalities in the state. The majority of deaths and years of life lost were recorded for females. The existence of priority areas and groups for adoption of prevention and mitigation policies stood out.

## Resumen

**Objetivo:**

Describir ocurrencias y daños humanos y materiales asociados a desastres naturales ocurridos en el estado de Río de Janeiro, Brasil, 2010-2022, así como estimar los años de vida perdidos por muertes resultantes de estos eventos.

**Métodos:**

Se trata de un estudio de cohorte prospectivo, con datos del Sistema Integrado de Información de Desastres y del Sistema de Información Hospitalarios del Sistema Único de Salud. Se realizaron análisis descriptivos de ocurrencias y daños humanos y materiales asociados a los desastres naturales seleccionados y se calcularon los años de vida perdidos para las muertes registradas bajo los códigos X36-39, por año, sexo y grupo de edad. **Resultados:** Ocurrieron 752 eventos naturales extremos, siendo la mayor frecuencia observada lluvias intensas (37,8%) y movimientos en masa (19,6%), seguidas de inundaciones (15,3%), inundaciones repentinas (15,2%), anegamiento (8,9%) y granizo. (3,3%). En cuanto a los daños humanos, se registraron 1.523 muertes, 3.379 heridos y 2.157 enfermedades. En cuanto a los daños materiales, se calcularon más de BRL 12 mil millones, siendo las viviendas, los establecimientos de salud y educación y la infraestructura urbana los más afectados. La mayoría de las muertes fueron mujeres (50,1%). El grupo de edad más afectado fue el de 15 a 59 años (51,8%). Se calcularon un total de 49.031,76 años de vida perdidos, de los cuales el 54,2% correspondieron al sexo femenino.

**Conclusión:**

La ocurrencia de desastres naturales y daños humanos y materiales afectaron, con mayor intensidad, sólo a unos pocos municipios del estado. La mayoría de las muertes y años de vida perdidos correspondieron a mujeres. Se destacó la existencia de áreas y grupos prioritarios para la adopción de políticas de prevención y mitigación.

**Ethical aspects**

This research respected ethical principles, having obtained the following approval data:

As this study used public domain data, without identifying the participants, it did not need to be submitted to a research ethics committee.

## Introduction

Climate changes seen in recent decades have contributed to the increase in natural disasters, which are becoming increasingly intense, causing economic, environmental and social losses ^1^. If no mitigation actions are taken, the climate crisis will increasingly threaten the health and livelihoods of people around the world, as well as ecosystem sustainability ^2^ ^)^ .

Goal 13 of the United Nations sustainable development goals pointed to the need to adopt urgent measures to combat climate change and its impacts, including by means of integrating measures to adapt to climate changes as part of national policies, strategies and planning. Goal 3 highlighted the need to manage health risks, which includes actions to prevent or minimize the risk of harm to population’s health due to adverse effects caused by the action of physical, chemical and biological agents, among others ^3^. Physical agents include natural disasters, which can cause illnesses or injuries, deaths, material damage, interruption of social and economic activity and environmental degradation ^4^.

In Brazil, several natural disasters are recorded every year, causing losses mainly in regions where more vulnerable groups live ^5^. Between 2013 and 2021, 51,184 disasters were recorded in the country, 50,481 of which were natural disasters and, most of them, climatological ^6^. Due to historical issues of unplanned urbanization and social inequality, poorer populations are more exposed and more vulnerable to such events ^7^ ^,^ ^8^.

Natural disasters are also known as extreme environmental events. These can be caused by natural phenomena of geological origin (earthquake, volcano, mass movement), hydrological (sudden and gradual flooding, pluvial flooding, mass movement/landslides), meteorological (tempest, storm, cyclone, gale), climatological (extreme temperatures, lack of rain, drought, forest fire, frost, hail), or biological events, which degrade both the natural and the built environment of the affected regions ^8^. Such events cause human, material and environmental damage that is often irreversible ^1^. It is clear that the vulnerability of the population has increased due to natural disasters ^9^.

In recent years, occurrence of natural disasters has increased in the state of Rio de Janeiro, causing a lot of damage. Between 2010 and 2022, 1,301 natural disasters were recorded ^10^. It should be noted that biological disasters associated with the COVID-19 pandemic were included in this total, but these were not considered in this research. 

It has been indicated that 81.5% of the state’s municipalities are susceptible to the occurrence of landslides, flash floods and flooding, affecting 5.7% of the population ^11^. In addition to greater frequency, the numbers have indicated that environmental disasters have intensified, with those of hydrological and geological origin being more common. Among these events, the landslides that occurred in Morro do Bumba (Niterói) in 2010 ^12^, in the state’s mountain region in 2011 ^13^ and in Petrópolis in 2022 ^14^ stood out, and caused major social, economic and environmental impacts. 

Adoption of integrated measures capable of mitigating such impacts depends, among other factors, on the analysis of these occurrences and their dynamics and impacts ^6^ ^,^ ^15^. The purpose of such analysis is to support the adoption of more effective mitigation policies, reducing losses and preserving, mainly, human life. Premature deaths caused by disasters affect society in different ways, with their impacts being felt in the short and long term.

The objective of this study was to describe the occurrences, human harm and material damage associated with natural disasters that occurred in the state of Rio de Janeiro. Brazil, between 2010 and 2022. Years of life lost due to deaths resulting from such events were also estimated.

## Methods

### Design

This was a prospective cohort study based on data on the occurrences of extreme natural disasters in the state of Rio de Janeiro, Brazil, between 2010 and 2022, as well as deaths associated with them.

### Background

The state of Rio de Janeiro has 92 municipalities, covers an area of 43,797.5 km² and borders with the states of Espírito Santo, Minas Gerais and São Paulo. In 2022, it had 16,055,174 inhabitants ^16^, the majority of whom lived in urban areas ^16^. A large part of its municipalities are located in areas susceptible to the occurrence of extreme natural disasters, making their populations more vulnerable to their impacts ^11^. 

### Participants

We selected records of deaths attributable to natural disasters under the following codes of the 10th Revision of the International Statistical Classification of Diseases and Related Health Problems (ICD-10): X36 (avalanche, landslide and other earth movements), X37 (cataclysmic storm), X38 (flood) and X39 (exposure to other forces of nature).

### Variables

The variables used in relation to deaths were the number of deaths from selected causes (ICD-10: X36-39), both total and by age group (under 1 year old, 1-4, 5-9, 10-14, 15-19, 20-24, 25-29, 30-34, 35-39, 40-44, 45-49, 50-54, 55-59, 60-64, 65-69, 70-74, 75-79, 80 or over), sex (male, female), year (2010-2022) and place of residence (municipality).

The variables used for the analysis of natural disasters were types of natural disaster, by location (municipality in the state of Rio de Janeiro) and date (day of occurrence in the period 2010-2022). Selection of disaster types followed the Brazilian Disaster Classification and Coding (Classificação e Codificação Brasileira de Desastres) guidelines. Given the greater risk of exposure and number of events in the past, the following types of disaster were considered: mass movements (geological), pluvial floods, flash floods and flooding (hydrological) and intense rainfall (climatological).

The variables used to represent human harm were the numbers of people who died, were ill, injured, homeless, displaced, missing or affected in other manners. The cost in monetary terms (in BRL) was considered for material damage: damage caused to housing units, health facilities, teaching facilities, service facilities, community facilities and infrastructure.

### Data collection

Data relating to deaths attributable to natural disasters for the period were extracted from the Brazilian National Health System (Sistema Único de Saúde) Information Technology Department website in June 2024 ^17^. These data were used to enable analysis of deaths by age group and sex (male and female), which made it possible to calculate years of life lost. 

The remaining data, related to occurrences, harm and damage caused in the period, were obtained from the Civil Defense Integrated Disaster Information System Sistema (Integrado de Informações de Desastres da Defesa Civil) ^18^. This system aggregated data recorded by municipalities that had filled out the Disaster Information Form indicating and quantifying the damage caused by the event. The monetary costs, in BRL, estimated for each occurrence were recorded on this Form.

Life expectancy was considered, according to Brazilian Institute of Geography and Statistics (Instituto Brasileiro de Geografia e Estatística) data, for each year covered by the research, by sex, in order to calculate life expectancy at the time of death ^19^. As there was no data that would enable analysis by age of each individual participant, we took the average of each age group available in the database. This average was subtracted from life expectancy by sex for each year of death, thus enabling the life expectancies used in the research to be calculated. 

### Data analysis

Absolute and relative frequencies were calculated for variables related to the number of natural disasters and human harm, by type, year and municipality. We calculated the sum, mean and standard deviation for human harm. The mean was calculated considering the frequency of harm, by type, and the total number of events in the period 2010-2022 (n=752). The number of deaths was calculated by year, municipality, sex and age group, thus enabling days of life lost to be calculated.

With regard to material damage, the sum of the monetary amounts recorded on the Disaster Information Form for each municipality, in BRL, was calculated for each occurrence recorded in the period 2010-2022. These amounts considered damaged or destroyed buildings and the estimated cost of the damage. Subsequently, the total amounts per year were calculated. These amounts were corrected for the year 2022 based on the General Price Index - Internal Availability (Índice Geral de Preços - Disponibilidade Interna) at the end of the period, thus enabling the total cost of the damage recorded to be calculated at 2022 prices.

Years of life lost were calculated as follows ^20^: years of life lost (c, i, s) = ∑ N (c, i, s) x E (i, s) ^1^, where N is the number of deaths attributable to cause c, for age i and sex s; and E is the life expectancy for age i and sex s, at the time of death. 

The data were collected from the aforementioned sources, aggregated according to the objectives and then tabulated. After preparing the spreadsheets, the calculations were performed using Microsoft Excel and SPSS version 29.0.

## Results

Between 2010 and 2022, 752 occurrences of natural disasters were recorded in the municipalities of the state of Rio de Janeiro. Intense rainfall (37.8%) and mass movements (19.6%) were most frequent, followed by floods (15.3%), flash floods (15.2%), pluvial flooding (8.9%) and hail (3.3%). The occurrences were related to several types of human harm, affecting 5,264,287 people (Table 1).

**Table 1:** Minimum and maximum values, sum, mean and standard deviation (SD) per number of recorded disasters, according to type of harm and total. State of Rio de Janeiro, Brazil, 2010-2022

Type of harm	Minimum; maximum	Sum	Mean ± SD
Dead	0; 420	1,523	2.03±22.49
Injured	0; 900	3,379	4.49±49.69
Ill	0; 1,033	2,157	2.87±41.46
Homeless	0; 8,288	48,427	64.40±461.51
Displaced	0; 25,853	272,875	362.87±1,598.47
Missing	0; 242	594	0.79±12.03
Affected in other manners	0; 750,535	4,935,332	6,562.94±39.167.28
Total	-	5,264,287	7,000.38±39,850.39

With regard to material damage, it affected the population in the short, medium and long term and resulted in unplanned public and private financial expenditure, which needed to be allocated in order to recover the destroyed areas (Table 2).

**Table 2:** Cost in BRL of material damage associated with the natural disasters selected, by type of building. State of Rio de Janeiro, Brazil. 2010-2022

Type of building	Value (BRL)
Housing units	7,751,909,818.70
Health facilities	77,236,920.67
Teaching facilities	185,228,237.62
Service facilities	3,823,020.77
Community facilities	26,710,785.35
Infrastructure	4,012,828,384.50
Total	12,057,737,167.61

**Figure 1:**
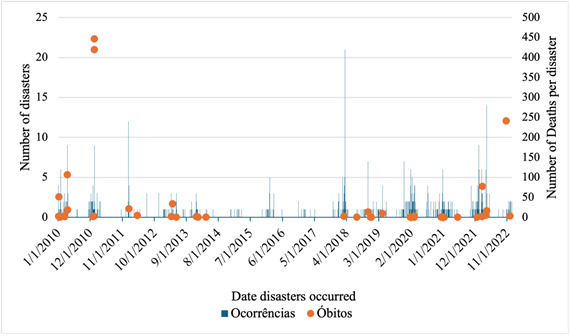
Occurrences of natural disasters and deaths by date. State of Rio de Janeiro, Brazil, 2010-2022 (n=1,521)

**Figure 2:**
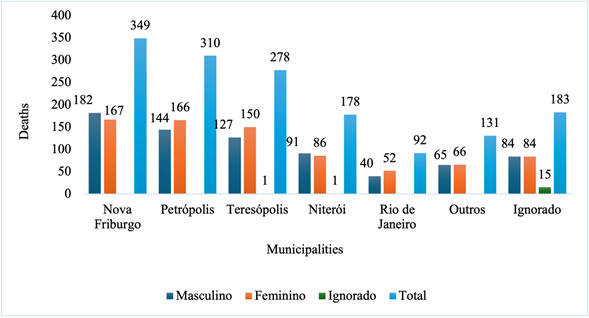
Deaths caused by natural disasters, by sex. State of Rio de Janeiro, Brazil, 2010-2022 (n=1,521)

Although there were 752 recorded occurrences, deaths were concentrated in certain events over time (Figure 1). The Brazilian National Health System Information Technology Department recorded 1,521 deaths (two less than the total recorded on the Integrated Disaster Information System) in the period 2010-2022, of which 50.1% were women (Figure 2).

The majority of deaths occurred in five municipalities in the state, notably Nova Friburgo (22.9%), Petrópolis (20.4%), Teresópolis (18.3%), Niterói (11.7%) and Rio de Janeiro (6.0%). The data indicated the need for adjustments to the way in which it is collected, as the municipality of residence of the disaster victim was not recorded for 12% of deaths.

The 15-59 age group was the most affected by deaths, accounting for 51.8% of total deaths recorded. Those under 14 years old accounted for 22%, and the elderly (over 60 years old) accounted for 15.6% of deaths. Ten percent of deaths were recorded with unknown age, another point that indicates the need to improve the process of collecting and recording information.

 In all, based on the methodology adopted for the period and area considered in this research, we calculated that 49,031.76 years of life were lost due to premature death, 54.2% of which related to females (Table 3).

**Table 3:** Years of life lost due to environmental disasters, by year, sex and total. State of Rio de Janeiro, Brazil, 2010-2022

Year	Male	Female	Total
2010	4,960.32	6,827.64	11,787.96
2011	13,561.24	15,421.80	28,983.04
2012	375.32	425	800.32
2013	1,023.25	898.16	1,921.41
2014	37.10	-	37.00
2015	-	-	-
2016	235.85	105	340.85
2017	58.00	-	58.00
2018	576.08	391.64	967.72
2019	121.64	262.19	383.83
2020	85.91	-	85.91
2021	64.54	-	64.54
2022	1,347.48	2,253.60	3,601.08
Total	22,446.73	26,585.03	49,031.76

## Discussion

The results indicated that natural disasters occurred in the state of Rio de Janeiro between 2010 and 2022, resulting in human harm and material damage, including deaths.

It stood out that recording occurrences and their impacts in more detail, with integration between information from the Civil Defense Integrated Disaster Information System and the Brazilian National Health System Information Technology Department, would contribute to more complete results on the issue dealt with by this research. The quality and organization of the data available enabled the proposed research objectives to be met.

Intense rainfall, pluvial flooding and flash floods were among the most frequent occurrences recorded. The states in the Southeast region of Brazil (where Rio de Janeiro is located) have been indicated as being more susceptible to risks of landslides, floods and flash floods ^21^, while the states in the country’s Northeast region have been more exposed to the occurrence of lack of rain and droughts ^22^ ^,^ ^23^.

The occurrences considered in this research generated impacts of different magnitudes, both human and material. Material losses are related to economic losses caused by damage to public and private properties, such as homes and facilities providing education and health services to the community, in addition to municipal infrastructure. 

The financial resources invested in the reconstruction of these facilities are one of the further impacts arising from the damage caused to services provided to the population, especially sensitive services such as education and health. In times of emergency, such as natural disasters, the provision of physical and mental health services is essential for maintaining the health and well-being of the population _(24)_. However, response capacity falls short of demand, bringing several negative impacts, especially for the most vulnerable ^25^. Spaces, affective relationships, dreams and identity deteriorate ^26^.

The most socially and economically vulnerable populations are those most exposed to risks. This indicates that the magnitude of these impacts depends not only on environmental factors, but also on economic and social factors, that is, on structural issues ^27^. 

Human harm refers to the number of deaths, injuries and illnesses, as well as displaced and homeless people. All these conditions impact families in different aspects and over time, requiring integrated action from public and non-governmental care services. 

Among the 1,521 deaths, the majority related to women. Despite the similar result in relation to the number of records by sex found in this research, women and girls are the most vulnerable in disaster situations, which are events that increase gender and racial inequalities ^28^ ^,^ ^29^ ^,^ ^30^. Due to lower purchasing power, high number of dependents and low access to environmental sanitation, women end up being exposed to greater risks, being historically and structurally more vulnerable ^29^ ^,^ ^30^. 

The data indicated that the number of female deaths was higher in the municipalities of Petrópolis, Teresópolis, Rio de Janeiro and in the other municipalities considered. Identifying areas with a higher concentration of more vulnerable people in terms of age and gender can contribute to defining relief and evacuation priorities in times of crisis. The 6-14 age group (16.0%) and the 15-59 age group (66.4%) lived in risk areas in the 476 municipalities analyzed by a study throughout Brazil ^21^. 

A national survey of people living in risk areas found that with regard to the 16 municipalities monitored in the state of Rio de Janeiro (17% of the state’s total municipalities), there were 2,937 risk areas with 865,000 people living in them. On average, 9.8% of the population was living in risk areas in the state of Rio de Janeiro, according to the municipalities monitored. São João de Meriti, Petrópolis, Teresópolis and Nova Friburgo stood out among these results. The three latter municipalities have experienced natural disasters with major impacts in recent years. In these municipalities, 24.4%, 28.0% and 18.5% of the population, respectively, was living in risk areas ^31^. 

Risk factors contribute to certain groups suffering greater impacts than others, because “socio-spatial segregation affects the different experiences of risk and the magnitude of the disaster, even when these populations, in different places in the city, are susceptible to the impacts of the same natural threat and the same hydrological and/or geological event” ^30^.

The calculation of years of life lost indicated higher levels for females. The number of days lost for females was 18% higher than that for males. This result can be explained by the higher number of deaths among women and the fact that they have a longer life expectancy than men, according to the data used in the research.

Years of life lost lead, among other factors, to loss of productivity and income in Brazil. Individuals who make up the economically active population (people aged 10 to 65 classified as employed or unemployed) cease working ^32^ ^,^ ^33^.

The results found showed that deaths and loss of years of life among the population were important impacts caused by the natural disasters considered in the research. This showed that the effects of climate change, together with the unequal socioeconomic structure existing in Brazil, can place the population at greater vulnerability. There is a need to adopt policies not only for risk management and urban planning, but also policies that contribute to improving the quality of life and well-being of the population, such as education, health and income policies.

## Data Availability

Not available.
