# Supplementary material for: Occurrences, human harm and years of life lost due to natural disasters in the state of Rio de Janeiro, Brazil, 2010-2022: a cohort study
Source: Epidemiol Serv Saude. 2025 Apr 18;34:e20240412. doi: 10.1590/S2237-96222025v34e20240412.en (PMC12014064; doi:10.1590/S2237-96222025v34e20240412.en)
Supplement: Translation

# Translation: Ocorrências, danos humanos e anos de vida perdidos por desastres naturais no estado do Rio de Janeiro, 2010-2022: estudo de coorte

**Authors:** Roberta Fernanda da Paz de Souza Paiva, Ana Luiza de Oliveira Maia, Juliana Beloti Giarola Martins

**Affiliations:** 1 Universidade Federal Fluminense, Escola de Engenharia Industrial Metalúrgica de Volta Redonda, Programa de Pós-Graduação em Tecnologia Ambiental, Volta Redonda, RJ, Brazil; 2 Universidade Federal Fluminense, Escola de Engenharia Industrial Metalúrgica de Volta Redonda, Engenharia de Agronegócios, Volta Redonda, RJ, Brazil

**Keywords:** Mudança Climática, Mortes Prematuras, Expectativa de Vida, Vulnerabilidade a Desastres, Estudos de Coortes.

## Abstract

**Objetivo:**

Descrever ocorrências e danos humanos e materiais associados aos desastres naturais ocorridos no estado do Rio de Janeiro, 2010-2022, bem como estimar os anos de vida perdidos devido aos óbitos resultantes desses eventos.

**Métodos:**

Trata-se de estudo de coorte prospectivo, com dados do Sistema Integrado de Informações sobre Desastres e do Sistemas de Informações Hospitalares do Sistema Único de Saúde. Realizaram-se análises descritivas de ocorrências e danos humanos e materiais associados aos desastres naturais selecionados e o cálculo dos anos de vida perdidos para os óbitos registrados sob os códigos X36-39, por ano, sexo e faixa etária.

**Resultados:**

Ocorreram 752 eventos naturais extremos, sendo a maior frequência observada para chuvas intensas (37,8%) e movimentos de massa (19,6%), seguidos por inundações (15,3%), enxurradas (15,2%), alagamentos (8,9%) e granizo (3,3%). Como danos humanos, registraram-se 1.523 mortos, 3.379 feridos e 2.157 enfermos. Quanto aos danos materiais, computaram-se mais de R$ 12 bilhões, sendo mais afetadas as unidades habitacionais, instalações de saúde e educação e infraestrutura urbana. A maioria dos óbitos foi do sexo feminino (50,1%). A faixa etária mais acometida foi a de 15 a 59 anos (51,8%). Calcularam-se 49.031,76 anos de vida perdidos, dos quais 54,2% registrados para o sexo feminino.

**Conclusão:**

As ocorrências e os danos humanos e materiais afetaram, de maneira mais intensa, poucos municípios do estado. A maioria dos óbitos e anos de vida perdidos foi registrada para o grupo feminino. Observou-se a existência de áreas e grupo prioritários para a adoção de políticas de prevenção e mitigação.

**Aspectos éticos**

A presente pesquisa respeitou os princípios éticos, obtendo os seguintes dados de aprovação:

Por utilizar dados de domínio público, sem a identificação dos indivíduos, não foi necessária a submissão do estudo a comitê de ética em pesquisa.

## Introdução

As mudanças climáticas observadas nas últimas décadas vêm contribuindo para o aumento dos desastres naturais, que se apresentam cada vez mais intensos, causando perdas econômicas, ambientais e sociais ^1^. Sem a adoção de ações de mitigação, a crise climática ameaça cada vez mais a saúde e os meios de subsistência das pessoas em todo o mundo, bem como a sustentabilidade dos ecossistemas ^2^.

A Organização das Nações Unidas, em seus objetivos do desenvolvimento sustentável, apontou, no objetivo 13, a necessidade de se adotarem medidas urgentes para o combate das alterações climáticas e seus impactos, por meio, entre outros, da integração de medidas de adaptação das mudanças do clima nas políticas, estratégias e planejamentos nacionais. No objetivo 3, destacou a necessidade de gerenciamento de riscos à saúde, que incluem ações para prevenir ou minimizar os riscos de danos à saúde da população devido aos efeitos adversos causados pela ação de agentes físicos, químicos, biológicos, entre outros ^3^. Os agentes físicos englobam os desastres naturais, podendo causar doenças ou agravos, óbitos, danos materiais, interrupção de atividade social e econômica e degradação ambiental ^4^.

No Brasil, registram-se anualmente diversos desastres naturais, acarretando perdas principalmente em regiões onde habitam grupos mais vulneráveis ^5^. Entre 2013 e 2021, foram registrados 51.184 desastres no país, sendo 50.481 desastres naturais e, em sua maioria, climatológicos ^6^. Devido às questões históricas de urbanização desordenada e desigualdade social, as populações mais pobres estão mais expostas e vulneráveis aos eventos ^7^ ^,^ ^8^.

Os desastres naturais também são conhecidos como eventos ambientais extremos. Estes podem ser causados por fenômenos naturais de origem geológica (terremoto, vulcão, movimento de massa), hidrológica (inundação brusca e gradual, alagamento, movimento de massa/deslizamentos), meteorológica (tempestade, tormenta, ciclone, vendaval), climatológica (temperatura extrema, seca, estiagem, incêndio florestal, geada, granizo), ou biológica, que degradam o ambiente natural e construído das regiões afetadas ^8^. Tais eventos provocam danos humanos, materiais e ambientais, muitas vezes irreversíveis ^1^. Observa-se que a vulnerabilidade da população aumentou devido aos desastres naturais ^9^.

Nos últimos anos, a ocorrência de desastres naturais aumentou no estado do Rio de Janeiro, acarretando muitos danos. Entre 2010 e 2022, registraram-se 1.301 desastres naturais ^10^. Ressalta-se que foram incluídos nesse total os desastres biológicos associados à pandemia de covid-19, os quais não foram considerados nesta pesquisa. 

Indicou-se que 81,5% dos seus municípios encontravam-se suscetíveis à ocorrência de deslizamentos, enxurradas e inundações, atingindo 5,7% da população ^11^. Além de maior frequência, os números indicavam que os desastres ambientais se intensificaram, sendo mais comuns os de origem hidrológica e geológica. Entre esses eventos, destacaram-se os deslizamentos de terra ocorridos no Morro do Bumba (Niterói) em 2010 ^12^, na região serrana em 2011 ^13^ e em Petrópolis em 2022 ^14^, que apresentaram grandes impactos sociais, econômicos e ambientais. 

A adoção de medidas integradas capazes de mitigar tais impactos depende, entre outros fatores, da análise dessas ocorrências e de sua dinâmica e seus impactos ^6^ ^,^ ^15^. Tem-se como finalidade embasar a adoção de políticas de mitigação mais efetivas, reduzindo as perdas e preservando, principalmente, a vida humana. As mortes prematuras ocasionadas pelos desastres afetam de diversas maneiras a sociedade, sendo seus impactos sentidos a curto e longo prazos.

O objetivo deste estudo foi descrever as ocorrências e os danos humanos e materiais associados aos desastres naturais ocorridos no estado do Rio de Janeiro entre 2010 e 2022. Estimaram-se também os anos de vida perdidos devido aos óbitos decorrentes de tais eventos.

## Métodos

### Desenho 

Tratou-se de estudo de coorte prospectivo baseado em dados sobre as ocorrências de desastres naturais extremos no estado do Rio de Janeiro, entre 2010 e 2022, e óbitos a eles associados.

### Contexo

O estado do Rio de Janeiro possui 92 municípios, ocupa a superfície de 43.797,5 km² e faz fronteira com Espírito Santo, Minas Gerais e São Paulo. Em 2022, a população era de 16.055.174 habitantes ^16^. A maioria destes residia na área urbana ^16^. Grande parte de seus municípios encontrava-se em áreas suscetíveis à ocorrência de desastres naturais extremos, tornando sua população mais vulnerável aos seus impactos ^11^. 

### Participantes

Foram selecionados os registros dos óbitos atribuíveis aos desastres naturais sob os códigos da 10ª Revisão da Classificação Estatística Internacional de Doenças e Problemas Relacionados à Saúde (CID-10): X36 (casos relativos a avalanche, desabamento de terra e outros movimentos da superfície terrestre), X37 (tempestade cataclísmica), X38 (inundação) e X39 (exposição a outras forças da natureza).

### Variáveis

As variáveis utilizadas com relação aos óbitos foram número de óbitos por causas selecionadas (CID-10: X36-39) total e segundo faixa etária (menor que 1 ano, 1-4, 5-9, 10-14, 15-19, 20-24, 25-29, 30-34, 35-39, 40-44, 45-49, 50-54, 55-59, 60-64, 65-69, 70-74, 75-79, 80 anos ou mais), sexo (masculino, feminino), ano (2010-2022) e local de residência (município).

As variáveis utilizadas para as análises dos desastres naturais foram tipos de desastres naturais, por local (município do estado do Rio de Janeiro) e data (dia da ocorrência no período 2010-2022). A seleção dos tipos de desastres obedeceu à Classificação e Codificação Brasileira de Desastres. Devido ao maior risco de exposição e números de eventos históricos, foram considerados os tipos de desastres: movimentos de massa (geológico), alagamentos, enchentes e inundações (hidrológico) e chuvas intensas (climatológico).

As variáveis utilizadas para representar os danos humanos foram os números de mortos, enfermos, feridos, desabrigados, desalojados, desaparecidos e de outros afetados. Considerou-se o valor monetário (em reais) para os danos materiais: dos danos acarretados às unidades habitacionais, instalações de saúde, instalações de ensino, instalações de serviço, instalações comunitárias e obras de infraestrutura. 

### Coleta de dados

Os dados relativos aos óbitos atribuíveis aos desastres naturais para o período foram extraídos do sítio eletrônico do Departamento de Informática do Sistema Único de Saúde em junho de 2024 ^17^. Esses dados foram utilizados por permitirem a análise dos óbitos por faixa etária e sexo (masculino e feminino), o que possibilitou que se calculassem os anos de vida perdidos. 

Os demais dados, relacionados às ocorrências e aos danos causados no período, foram obtidos no Sistema Integrado de Informações de Desastres da Defesa Civil ^18^. Esse sistema agregava os dados registrados pelos municípios, que preenchiam o Formulário de Informações de Desastres indicando e quantificando os danos causados pela ocorrência do evento. Nesse formulário foram registrados os valores monetários, em reais, estimados para cada ocorrência. 

Considerou-se a expectativa de vida, segundo o Instituto Brasileiro de Geografia e Estatística para cada ano considerado na pesquisa, por sexo, para o cálculo da expectativa de vida à época do óbito ^19^. Como não havia dados que permitissem a análise por idade de cada indivíduo, foi considerada a média de cada faixa etária disponibilizada no banco de dados. Essa média foi subtraída da expectativa de vida por sexo para cada ano de óbito, permitindo que as expectativas de vida utilizadas na pesquisa fossem calculadas. 

### Análise de dados

Calcularam-se frequências absolutas e relativas para as variáveis relacionadas ao número de desastres naturais e aos danos humanos, por tipo, ano e município. Para os danos humanos, foram calculados a soma, a média e o desvio padrão. O cálculo da média considerou a frequência de danos, por tipo, e o total de eventos no período 2010-2022 (n=752). O número de óbitos foi calculado por ano, município, sexo e faixa etária, permitindo que os dias de vida perdidos fossem calculados. 

Para os danos materiais, calculou-se a soma dos valores registrados no Formulário de Informações dos Desastres por cada município, em reais, para cada ocorrência registrada no período 2010-2022. Esses valores consideraram as edificações danificadas ou destruídas e o valor estimado do dano. Posteriormente, foram calculados os totais dos valores por ano. Esses valores foram corrigidos para o ano de 2022 com base no Índice Geral de Preços - Disponibilidade Interna do final do período, permitindo o cálculo do total dos danos registrados, em valores de 2022. 

Os anos de vida perdidos foram calculados da seguinte forma ^(20)^: anos de vida perdidos (c, i, s) = ∑ N (c, i, s) x E (i, s) ^1^, em que N é o número de óbitos atribuíveis à causa c, para idade i e sexo s; e E é a expectativa de vida para a idade i e o sexo s, à época do óbito. 

Os dados foram coletados nos bancos das fontes citadas, agregados de acordo com os objetivos e tabulados. Após a elaboração das planilhas, os cálculos foram realizados com o auxílio dos softwares Microsoft Excel e SPSS versão 29.0. 

## Resultados

Entre 2010 e 2022, foram registradas 752 ocorrências de desastres naturais nos municípios do Rio de Janeiro. As chuvas intensas (37,8%) e os movimentos de massa (19,6%) apresentaram maior frequência, seguidos por inundações (15,3%), enxurradas (15,2%), alagamentos (8,9%) e granizo (3,3%). As ocorrências estão relacionadas aos diversos danos humanos, afetando 5.264.287 pessoas (Tabela 1).

**Tabela 1:** Valores mínimos e máximos, soma, média e desvio padrão (DP) pelo número de desastres registrados, segundo tipo de dano e total. Estado do Rio de Janeiro, 2010-2022

Tipo de dano	Mínimo; máximo	Soma	Média ± DP
Mortos	0; 420	1.523	2,03±22,49
Feridos	0; 900	3.379	4,49±49,69
Enfermos	0; 1.033	2.157	2,87±41,46
Desabrigados	0; 8.288	48.427	64,40±461,51
Desalojados	0; 25.853	272.875	362,87±1.598,47
Desaparecidos	0; 242	594	0,79±12,03
Outros afetados	0; 750.535	4.935.332	6.562,94±39.167,28
Total	-	5.264.287	7.000,38±39.850,39

Outros danos foram os materiais. Estes afetaram a população no curto, médio e longo prazos e acarretaram prejuízos monetários públicos e privados, que precisaram ser aplicados para recuperação das áreas destruídas (Tabela 2).

**Tabela 2:** Custo dos danos materiais associados aos desastres naturais selecionados em reais (R$) por tipo de edificação. Estado do Rio de Janeiro, 2010-2022

Tipo de edificação	Valor (R$)
Unidades habitacionais	7.751.909.818,70
Instalações de saúde	77.236.920,67
Instalações de ensino	185.228.237,62
Instalações de serviço	3.823.020,77
Instalações comunitárias	26.710.785,35
Obras de infraestrutura	4.012.828.384,50
Total	12.057.737.167,61

Apesar das 752 ocorrências registradas, os óbitos foram concentrados em determinados eventos ao longo do tempo (Figura 1). O Departamento de Informática do Sistema Único de Saúde registrou 1.521 óbitos (dois a menos que o total registrado no Sistema Integrado de Informações sobre Desastres) no período 2010-2022, dos quais 50,1% foram mulheres (Figura 2).

**Figura 1:** Ocorrências de desastres naturais e óbitos por data. Estado do Rio de Janeiro, 2010-2022 (n=1.521)

**Figura 2:** Óbitos ocasionados por desastres naturais, por sexo. Estado do Rio de Janeiro, 2010-2022 (n=1.521)

Os óbitos ocorreram, em sua maioria, em cinco municípios do estado, destacando-se Nova Friburgo (22,9%), Petrópolis (20,4%), Teresópolis (18,3%), Niterói (11,7%) e Rio de Janeiro (6,0%). Os dados indicaram a necessidade de ajustes na forma de coleta, já que não foi registrado o município de residência da vítima do desastre para 12% dos óbitos.

A faixa etária mais acometida pelos óbitos foi a de 15 a 59 anos, na qual foram registrados 51,8% do total. Os menores de 14 anos corresponderam a 22%, e os idosos (acima de 60 anos), a 15,6% dos óbitos. Dez por cento dos óbitos estavam registrados com idade ignorada, outro ponto que indicou a necessidade de melhoria no processo de coleta e registro das informações.

No total, a partir da metodologia adotada para o período e a área considerados nesta pesquisa, foram calculados 49.031,76 anos de vida perdidos devido aos óbitos precoces, dos quais 54,2% foram registrados para o sexo feminino (Tabela 3).

**Tabela 3:** Anos de vida perdidos em decorrência de desastres ambientais, por ano, sexo e total. Estado do Rio de Janeiro, 2010-2022

Ano	Masculino	Feminino	Total
2010	4.960,32	6.827,64	11.787,96
2011	13.561,24	15.421,80	28.983,04
2012	375,32	425	800,32
2013	1.023,25	898,16	1.921,41
2014	37,10	-	37,00
2015	-	-	-
2016	235,85	105	340,85
2017	58,00	-	58,00
2018	576,08	391,64	967,72
2019	121,64	262,19	383,83
2020	85,91	-	85,91
2021	64,54	-	64,54
2022	1.347,48	2.253,60	3.601,08
Total	22.446,73	26.585,03	49.031,76

## Discussão

Os resultados indicaram que ocorreram desastres naturais no estado do Rio de Janeiro entre 2010 e 2022, dos quais resultaram danos humanos e materiais, incluindo óbitos.

Destacou-se que o registro das ocorrências e seus impactos de maneira mais detalhada, com integração entre as informações do Sistema Integrado de Informações de Desastres da Defesa Civil e do Departamento de Informática do Sistema Único de Saúde, contribuiria para resultados mais completos acerca da problemática tratada na pesquisa. A qualidade e a organização dos dados disponíveis permitiram que fossem atendidos os objetivos propostos.

As chuvas intensas, os alagamentos e as enchentes foram registrados entre as ocorrências mais frequentes. Indicaram-se os estados da região Sudeste como mais suscetíveis aos riscos de deslizamentos, inundações e enxurradas ^21^. Os estados da região Nordeste estavam mais expostos à ocorrência de estiagens e secas ^22^ ^,^ ^23^.

As ocorrências consideradas nesta pesquisa geraram impactos de diferentes magnitudes, sendo humanos e materiais. As perdas materiais estão relacionadas aos prejuízos econômicos acarretados pelos danos aos imóveis públicos e privados, tais como os domicílios e as instalações de prestação de serviços de educação e saúde à comunidade, além da infraestrutura municipal. 

Os recursos financeiros investidos na reconstrução dessas instalações apresentam-se como um dos impactos que se soma ao prejuízo dos serviços prestados à população, principalmente serviços sensíveis como a educação e a saúde. Em momentos de emergência, como o caso de desastres naturais, a prestação dos serviços de saúde física e mental é fundamental para a manutenção da saúde e do bem-estar da população ^24^. A capacidade de resposta fica aquém da demanda, trazendo diversos impactos negativos, principalmente aos mais vulneráveis ^25^. Deterioram-se, assim, espaços, relações afetivas, sonhos, identidade ^26^.

As populações mais vulneráveis social e economicamente são as mais expostas aos riscos. Isso indica que a magnitude desses impactos depende não apenas de fatores ambientais, mas também econômicos e sociais, ou seja, de questões estruturais ^27^. 

Os danos humanos indicam número de mortos, feridos e enfermos, além de desalojados e desabrigados. Todas essas condições impactam as famílias sob diversos aspectos e espaços de tempo, demandando ação integrada dos serviços públicos e não governamentais de atendimento. 

Entre os 1.521 óbitos, a maioria foi de mulheres. Apesar do resultado semelhante em relação ao número de registros por sexo encontrado nesta pesquisa, as mulheres e meninas são as mais vulneráveis em situações de desastres, que são eventos que potencializam as desigualdades de gênero e raça ^28^ ^,^ ^29^ ^,^ ^30^. Devido ao menor poder aquisitivo, ao elevado número de dependentes e ao baixo acesso ao saneamento ambiental, as mulheres acabam sendo expostas aos maiores riscos, sendo histórica e estruturalmente mais vulneráveis ^29^ ^,^ ^30^. 

Os dados indicaram que o número de óbitos de mulheres foi maior nos municípios de Petrópolis, Teresópolis, Rio de Janeiro e nos outros considerados. A determinação de áreas com maior concentração de pessoas mais vulneráveis quanto a idade e sexo pode contribuir para a definição de prioridades de socorro e evacuação em momento de crises. As faixas etárias de 6 a 14 anos (16,0%) e de 15 a 59 anos de idade (66,4%) ocupavam áreas de risco nos 476 municípios analisados em todo Brasil ^21^.

Ao levantar a população do país residente em áreas de risco, considerando-se os 16 municípios monitorados no estado do Rio de Janeiro (17% do total de municípios do estado), foram encontradas 2.937 áreas de risco e 865 mil pessoas residentes nessas áreas. A média de habitantes que residem em áreas de risco no estado, segundo os municípios monitorados, foi 9,8%. São João de Meriti, Petrópolis, Teresópolis e Nova Friburgo mereceram destaque nesses resultados. Estes três últimos passaram por desastres naturais com grandes impactos nos últimos anos. Nesses municípios, a população residente em áreas de risco era 24,4%, 28,0% e 18,5% ^31^. 

Os fatores de risco contribuem para que determinados grupos sofram maiores impactos que outros, isto porque “a segregação socioespacial afeta as distintas vivências do risco e a magnitude do desastre, mesmo quando essas populações, em lugares diferentes na cidade, estejam suscetíveis aos impactos da mesma ameaça natural e ao mesmo evento hidrológico e/ou geológico” ^30^.

O cálculo dos anos de vida perdidos indicou maiores resultados para o sexo feminino. O número de dias perdidos para o sexo feminino foi 18% superior ao dado para o sexo masculino. Esse resultado pode ser explicado pelo maior número de óbitos entre as mulheres e pelo fato de essas apresentarem expectativa de vida maior que os homens, de acordo com os dados utilizados na pesquisa.

Os anos de vida perdidos acarretam, entre outros fatores, à perda de produtividade e renda do país. Os indivíduos componentes da população economicamente ativa (pessoas de 10 a 65 anos classificadas como ocupadas ou desocupadas) deixaram de ser conduzidos aos postos de trabalho ^32^ ^,^ ^33^.

Os resultados encontrados mostraram que os óbitos e a perda de anos de vida da população apresentaram-se como importantes impactos acarretados pelos desastres naturais considerados na pesquisa. Isso mostrou que os efeitos das mudanças climáticas, em conjunto à estrutura socioeconômica desigual observada no Brasil, pode trazer maior vulnerabilidade à população. Indica-se a necessidade da adoção de políticas não apenas de gestão de riscos e planejamento urbano, mas também daquelas que contribuam para a melhoria da qualidade de vida e do bem-estar da população, como as educacionais, de saúde e de renda.

## Data Availability

Os dados da pesquisa não estão disponíveis.
